# A possible case of bictegravir-associated severe unconjugated hyperbilirubinemia

**DOI:** 10.1186/s12981-023-00501-7

**Published:** 2023-01-23

**Authors:** Kanak Parmar, Poemlarp Mekraksakit, Kenneth Nugent, Jacob Nichols

**Affiliations:** 1 Department of Internal Medicine, Texas Tech University Health Sciences Center, 3601 4th St, Lubbock, TX USA; 2 Department of Pulmonary and Critical Care, Texas Tech University Health Sciences Center, Lubbock, TX USA; 3 Division of Infectious Disease, Texas Tech University Health Sciences Center, Lubbock, TX USA

**Keywords:** Hyperbilirubinemia, Biktarvy, Bictegravir

## Abstract

**Background:**

Bictegravir (BIC) co-formulated with emtricitabine (FTC) and tenofovir alafenamide (TAF) is approved by Federal Food and Drug Administration in 2018 for both treatment-naïve and experienced persons living with HIV (PLWH).

**Case presentation:**

A young man with recently diagnosed human immunodeficiency virus (HIV) infection presented with jaundice. Blood work was significant for mild anemia and grade 4 unconjugated hyperbilirubinemia. A comprehensive evaluation for hemolytic anemia failed to reveal any etiology. Other causes of hyperbilirubinemia were negative. Four months prior, patient was started on antiretroviral therapy with a single tablet regimen containing bictegravir/emtricitabine/tenofovir alafenamide (BIC/FTC/TAF), brand name Biktarvy^®^, and the medication was suspected to be the cause. The medication was held, and the hyperbilirubinemia improved.

**Conclusion:**

Severe hyperbilirubinemia can be found in the patient using BIC/FTC/TAF. The data for this adverse reaction is scarce, and more studies are needed on this possible side effect. The mechanism of unconjugated hyperbilirubinemia by INSTI remains undefined.

## Background

There are more than 30 antiretroviral medications (ARV) currently available for the treatment of HIV. These primarily belong to five major classes: nucleoside (and nucleotide) reverse transcriptase inhibitors (NRTIs), non-nucleoside reverse transcriptase inhibitors (NNRTI), protease inhibitors (PIs), integrase strand transfer inhibitors (INSTIs) and entry inhibitors. Bictegravir (BIC), is co-formulated with emtricitabine (FTC) and tenofovir alafenamide (TAF) and is approved by Federal Food and Drug Administration in 2018 for both treatment-naïve and experienced persons living with HIV (PLWH) [1]. Subsequently, the Department of Health and Human Services (DHHS) Panel on Antiretroviral Guidelines for Adults and Adolescents in 2018 as well as the International Antiviral Society (IAS) in 2020 has recommended BIC/FTC/TAF as one of the first line medications in treatment for PLWH [2, 3].

Antiretroviral medications are known to cause adverse effects on liver including idiosyncratic reactions, direct cholestatic injury, mitochondrial dysfunction, nonalcoholic steatohepatitis (NASH), or isolated hyperbilirubinemia [4, 5]. In the two clinical trials in adults with no previous antiretroviral treatment, BIC/FTC/TAF caused hyperbilirubinemia in 12% of subjects [6]. The increase in bilirubin was primarily Grade 1, i.e., 1–1.5X ULN (upper limit of normal) in 9% of subjects and Grade 2, i.e., 1.5 to 2.5 X ULN in 3% of subjects [7]. Here we report a case of a healthy man who presented with grade 4 hyperbilirubinemia (> 5X ULN).

## Case presentation

An eighteen-year-old man with medical history of recently diagnosed HIV infection presented with epigastric pain, nausea, and vomiting for 1 week. He denied any history of recent travel, infection, or close contact with a sick person. Social history was negative for alcohol, tobacco, or illicit drug use. The patient denied any other herbal or over-the-counter medications. Surgical history was negative. Family history was negative for any inherited disorders. He was started on BIC/FTC/TAF four months prior. Physical examination revealed jaundice and scleral icterus with anterior cervical lymphadenopathy. No abdominal tenderness or hepatosplenomegaly was noted.

## Investigations

The laboratory results revealed normochromic normocytic anemia, unconjugated hyperbilirubinemia with total and indirect bilirubin concentrations of 17.2 and 16.2 mg/dL respectively (Table 1). Baseline laboratory values showed mild elevation in bilirubin concentrations. Liver enzymes were normal before as well as on presentation. Synthetic function of liver was normal. Evaluation for hemolytic anemia showed elevated reticulocyte count and decreased haptoglobin concentrations. Lactate dehydrogenase was normal. The peripheral blood smear showed minimal spherocytosis and macrocytosis. However, the direct antiglobulin test (DAT) and glucose-6-phosphate dehydrogenase (G-6-PD) screen were negative. Vitamin B12 and folate levels were normal. His last CD4 count was 469 cells/mm^3^ and viral load was 93 copies/ml three months prior to admission. Abdominal ultrasound did not reveal any abnormalities.

**Table 1:** Laboratory values

	Three months before admission	On admission	Day 3	3 weeks later	2 months later	Reference values
WBC (K/uL)	5.20	7.41	6.46	6.82	6.5	4.5–5.2
Hemoglobin (g/dl)	15.5	16.1	12.1	11.7	16.3	13–16
Platelets (K/uL)	194	188	163	166	157	163–667
MCV (fl)	90.1	89.9	101.4	102	95.6	78–98
Reticulocyte %			4.83			0.5–1.8
Total bilirubin (mg/dL)	2.1	17.2	11.2	4.3	2.8	0–1
Direct bilirubin (mg/dL)	0.3	1	0.5	0.2		0–0.2
Indirect bilirubin (mg/dL)		16.2	10.7	4.1		0.1–1.1
ALP (IU/L)	81	55	45	63	70	35–129
ALT (IU/L)	16	16	18	13	39	5–41
AST (IU/L)	19	19	18	18	33	5–37
LDH (U/L)		205				135–225
Folate (ng/ml)		7.4				
TSH (mcIU/ml)		0.64				0.27–4.2
Vitamin B12 (pg/ml)		303				232–1425
HIV RNA PCR copies/ml	93				426	
CD4%	32				21	31–59
CD8%	44				53	13–37
ABO/Rh		O positive				
Haptoglobin (mg/dL)		< 8				43–212
Hepatitis A Virus,^©^ Ig G		Reactive				
Hepatitis A Virus, IgM		Non-Reactive				
Hepatitis B Surface Ag		Non-Reactive				
Hepatitis B Surface Ab		Positive				
Hepatitis B Core Ab IgG		Non-Reactive				
Hepatitis C Ab IgG		Non-Reactive				

*MCV* mean corpuscular volume, *ALP* alkaline phosphatase, *ALT* alanine transaminase, *AST* aspartate transaminase, *LDH* lactate dehydrogenase, *TSH* thyroid stimulating hormone, *RNA* ribonucleic acid, *PCR* polymerase chain reaction, *Rh* rhesus, *CD* cluster of differentiation, *Ab* antibody, *Ig* immunoglobulin

## Differential diagnosis

Based on history, physical examination, and initial laboratory results, the patient needed evaluation for this degree of unconjugated hyperbilirubinemia. Medication side effect was the most likely cause of hyperbilirubinemia given recent history of taking new medication. Naranjo score calculated was 3 indicating possible adverse drug reaction [8]. He did not have elevation in hepatic enzymes or abnormal findings on abdominal ultrasonography which rule out cholestatic injury and obstructive jaundice. The differential diagnosis included overproduction of bilirubin due to hemolytic anemia given undetectable haptoglobin concentration, reticulocytosis. However, the patient did not have any anemia on admission. Testing for autoimmune hemolytic anemia and G-6-PD deficiency were also negative. Spherocytosis was also minimal in a blood smear. Congenital hemolytic evaluation was not obtained due to low likelihood. The differential diagnosis also included pathologies involving decreased uptake of bilirubin by liver, such as Gilbert’s syndrome, or defects with conjugation, example Criggler Najjar. Medication side effect was also a possible etiology.

## Treatment

Due to concern for medication side effect as the cause of the hyperbilirubinemia, BIC/FTC/TAF was held on the day of admission. The bilirubin started to trend down to bilirubin 11.2 mg/dL. Patient’s nausea and pain resolved. He was discharged dolutegravir and lamivudine, another STR on day 3 with no increase in bilirubin on follow up labs.

## Outcome and follow-up

Three weeks after discharge, the total bilirubin concentrations were 4.3 mg/dL and further improved to 2.8 mg/dL at two months.

## Discussion

BIC/FTC/TAF has been the first line treatment for PLWH and shown to be non-inferior to other STR containing dolutegravir in two clinical trials [7, 9]. GS-US-380-1490, a randomized clinical trial (RCT) tested BIC/FTC/TAF against dolutegravir, emtricitabine and tenofovir alafenamide and showed lower incidence of drug related events in the bictegravir group [7]. Another RCT GS-US-380–1489 compared BIC/FTC/TAF against dolutegravir, abacavir and lamivudine reported lower drug-related adverse events and two times lower in vitro resistance in the bictegravir group [9]. The adverse events in the dolutegravir group were primarily driven by nausea [9].

The contemporary ARV drugs have been associated with adverse events on liver by various mechanisms [4]. For example, the NNRTI have been shown in clinical trials to cause grade 3/ 4 elevations in alanine transaminase (ALT) / aspartate transaminase (AST) by causing direct cholestatic injury, hypersensitivity, or the immune reconstitution syndrome [10]. However, doravirine, an NNRTI has been shown to cause grade 2 bilirubin elevation in 2% patients enrolled in DRIVE-FORWARD trial without any other liver function test abnormalities [11].

The hepatotoxicity associated with NRTIs is thought to be mediated by mitochondrial toxicity [5]. PIs also carry warning for elevations in ALT/AST in preexisting liver disease and can cause acute hepatitis [4]. However, atazanavir (ATV), a PI is known to cause reversible increase in unconjugated bilrubin without liver injury due to UGT1A1 inhibition. In CASTLE study, ATV with ritonavir (ATV/r) was compared against lopinavir /ritonavir (LPV/r). It showed that 44% patients receiving ATV/r developed hyperbilirubinemia at any time through 96 weeks with 5% developing jaundice and < 1% discontinued treatment due to hyperbilirubinemia. It was not associated with abnormalities in liver transaminases or hepatic function [12].

Bilirubin is the product of heme catabolism. It is cleared by the liver by converting into a water-soluble metabolite for secretion into bile. This is done by conjugating bilirubin with glucuronic acid by bilirubin uridine diphosphate-glucuronosyltransferase (UGT), a microsomal enzyme [13].Unconjugated hyperbilirubinemia can be caused by either decreased uptake of bilirubin by liver cells or a defect in conjugation. Protease inhibitors (PIs) such as indinavir and ATV have an inhibitory effect on UGT enzyme and can cause hyperbilirubinemia, a mechanism similar to Gilbert syndrome [14]. Genetic polymorphisms of *UGT1A1* gene have been known to impact UGT activity along with polymorphisms in multidrug resistance gene1(MDR1), a transporter for ATV inside cells. Haplotype *UGT1A1, UGT1A3, UGT1A7* and MDR1 3435 have been associated with hyperbilirubinemia [14]. PIs also have been reported to inhibit the human organic anion transporting protein 1B1, which transports unconjugated bilirubin to the liver [15].

The hyperbilirubinemia by INSTIs is rarely reported and the mechanism remains unclear [4]. In a phase III trial with the new medication cabotegravir combined with rilpivirine, minor increase in bilirubin concentrations were noted and thought to be due to unconjugated bilirubin competing for UGT1A1 [16]. This may be a similar mechanism of unconjugated hyperbilirubinemia from BIC/FTC/TAF but it remains unknown.

The common adverse events reported in the Trial 1489 and 1490 for BIC/FTC/TAF were diarrhea, nausea, headache, fatigue, abnormal dreams, dizziness and insomnia in > 2% of HIV-1 infected patients [6]. More than 2% of the patients had grade 3 and 4 laboratory abnormalities such as elevation of hepatic enzymes, neutrophils, and cholesterol concentrations. Hyperbilirubinemia was seen in 12% of subjects in trials but was Grade 1 and Grade 2(6). Our case presented with grade 4 hyperbilirubinemia leading to discontinuation of the medication which has not been reported previously.

Among the medications in BIC/FTC/TAF, FTC and TAF are primarily excreted renally; the excretion of BIC is primarily hepatic. BIC is substrate for cytochrome P450-3A4 (CYP3A4) and uridine diphosphate glucuronosyltransferase 1 family, polypeptide A1 (UGT1A1), but the mechanism of hyperbilirubinemia, though looks similar to cabotegravir, remains to be defined [14]. In our case the laboratory value was significant for low haptoglobin level which might suggest a concomitant hemolytic process. There is scarce data on antiretroviral drugs effects on erythrocyte’s cell membrane in vivo and clinical data is limited to case reports [17, 18].

It might be possible that our patient had an underlying defect in bilirubin metabolism, for example Gilbert's syndrome and an additional inhibition of the enzyme by medication can presented with florid hyperbilirubinemia.

## Conclusion

This case presented with grade 4 hyperbilirubinemia while on BIC/FTC/TAF leading to discontinuation of the medication. The data for this adverse reaction is scarce, and more studies are needed on this possible side effect.

## Learning points

- Hyperbilirubinemia (Grade 1 and Grade2) has been found in 12% patients in trials with BIC/FTC/TAF. Severe hyperbilirubinemia can be found in the patient using BIC/FTC/TAF.
- The mechanism of unconjugated hyperbilirubinemia in PIs is primarily through inhibition of UDP-glucuronosyltransferases (UGT). However, the mechanism by INSTI remains undefined.
- In cases where patients develop severe hyperbilirubinemia from HIV ARV therapy, an underlying Gilbert syndrome should be investigated.

## Data Availability

Not applicable.

## References

[1] https://www.gilead.com/news-and-press/press-room/press-releases/2018/2/us-food-and-drug-administration-approves-gileads-biktarvy-bictegravir-emtricitabine-tenofovir-alafenamide-for-treatment-of-hiv1-infection. Accessed 7 Feb 2018.

[2] https://www.natap.org/2018/HIV/032818_01.htm.

[3] Saag MS, Gandhi RT, Hoy JF, Landovitz RJ, Thompson MA, Sax PE (2020). Antiretroviral drugs for treatment and prevention of HIV infection in adults: 2020 recommendations of the international antiviral society-USA panel. JAMA.

[4] Otto AO, Rivera CG, Zeuli JD, Temesgen Z (2021). Hepatotoxicity of contemporary antiretroviral drugs: a review and evaluation of published clinical data. Cells.

[5] Neff GW, Jayaweera D, Sherman KE (2006). Drug-Induced liver injury in HIV patients. Gastroenterol Hepatol.

[6] Biktarvy (bictegravir, emtricitabine, tenofovir alafenamide) [prescribing information]. Foster City, CA: Gilead sciences March 2021.

[7] Sax PE, Pozniak A, Montes ML, Koenig E, DeJesus E, Stellbrink HJ (2017). Coformulated bictegravir, emtricitabine, and tenofovir alafenamide versus dolutegravir with emtricitabine and tenofovir alafenamide, for initial treatment of HIV-1 infection (GS-US-380-1490): a randomised, double-blind, multicentre, phase 3, non-inferiority trial. Lancet.

[8] Naranjo CA, Busto U, Sellers EM, Sandor P, Ruiz I, Roberts EA (1981). A method for estimating the probability of adverse drug reactions. Clin Pharmacol Ther.

[9] Gallant J, Lazzarin A, Mills A, Orkin C, Podzamczer D, Tebas P (2017). Bictegravir, emtricitabine, and tenofovir alafenamide versus dolutegravir, abacavir, and lamivudine for initial treatment of HIV-1 infection (GS-US-380-1489): a double-blind, multicentre, phase 3, randomised controlled non-inferiority trial. Lancet.

[10] Sulkowski MS, Thomas DL, Mehta SH, Chaisson RE, Moore RD (2002). Hepatotoxicity associated with nevirapine or efavirenz-containing antiretroviral therapy: role of hepatitis C and B infections. Hepatology.

[11] Molina JM, Squires K, Sax PE, Cahn P, Lombaard J, DeJesus E (2020). Doravirine versus ritonavir-boosted darunavir in antiretroviral-naive adults with HIV-1 (DRIVE-FORWARD): 96-week results of a randomised, double-blind, non-inferiority, phase 3 trial. Lancet HIV.

[12] McDonald C, Uy J, Hu W, Wirtz V, Juethner S, Butcher D (2012). Clinical significance of hyperbilirubinemia among HIV-1-infected patients treated with atazanavir/ritonavir through 96 weeks in the CASTLE study. AIDS Patient Care STDs.

[13] Erlinger S, Arias IM, Dhumeaux D (2014). Inherited disorders of bilirubin transport and conjugation: new insights into molecular mechanisms and consequences. Gastroenterology.

[14] Tozzi V (2010). Pharmacogenetics of antiretrovirals. Antiviral Res.

[15] Campbell SD, de Morais SM, Xu JJ (2004). Inhibition of human organic anion transporting polypeptide OATP 1B1 as a mechanism of drug-induced hyperbilirubinemia. Chem Biol Interact.

[16] Rizzardini G, Overton ET, Orkin C, Swindells S, Arasteh K, Górgolas Hernández-Mora M (2020). Long-acting injectable cabotegravir + rilpivirine for HIV maintenance therapy: week 48 pooled analysis of phase 3 ATLAS and FLAIR trials. J Acquir Immune Defic Syndr.

[17] Bissinger R, Waibel S, Lang F (2015). Induction of suicidal erythrocyte death by nelfinavir. Toxins.

[18] Watson A (2000). Reversible acute haemolysis associated with indinavir. AIDS.

